# IL-6 after wake-up in human males: Exposure to red versus blue light and the interplay with cortisol

**DOI:** 10.1016/j.bbih.2024.100833

**Published:** 2024-08-02

**Authors:** Liza Mekschrat, Bjarne Schmalbach, Nicolas Rohleder, Katja Petrowski

**Affiliations:** a Medical Psychology & Medical Sociology, University Medical Center of the Johannes Gutenberg University Mainz, Mainz, Germany; b Department of Psychology, Friedrich-Alexander-Universität Erlangen-Nürnberg, Erlangen, Germany

**Keywords:** Light exposure, Cortisol, Cortisol awakening response, Circadian rhythm, Inflammatory immune system, IL-6, Cortisol cytokine interplay, Sleep

## Abstract

Light is essential in shaping human circadian rhythms, including that of the hormone cortisol. While cortisol is known to influence secretion of the cytokine IL-6, the influence of light itself on IL-6 remains unclear. Thus, this study investigated the effects of two light conditions – red and blue – on IL-6 concentrations and the cortisol awakening response in blood. The interplay between cortisol and IL-6 was explored as well. The between-subject experiment was conducted with 71 healthy adult men (aged *M*_red_ = 24.30, *SD* = 3.56; *M*_blue_ = 24.40, *SD* = 3.51) in a standardized sleep laboratory setting with 60-min light exposure post-awakening at 05:00 a.m. Two mixed models, with light condition and time across measurement points as factors, were calculated. In the one for cortisol, chronotype was introduced as a covariate. Mean cortisol concentrations did not differ between exposure to red vs. blue light (*p* = 0.443), but overall cortisol output (area under the curve with respect to ground; AUC_G_) and sensitivity (area under the curve with respect to increase; AUC_I_) were greater in the blue-light condition (*p* = 0.050 and *p* < 0.001, respectively). Additionally, chronotype significantly influenced cortisol concentrations (*p* = 0.035). As for IL-6, a main effect of time was obtained, with increasing concentrations over time (*p* = 0.002). Total IL-6 secretion was greater under blue-light exposure (*p* <. 001), but mean IL-6 concentrations (*p* = 0.230) and IL-6 sensitivity (*p* = 0.777) did not differ between the red- and blue-light condition. Mean and total cortisol and IL-6 concentrations were significantly negatively correlated (*p* = 0.021 and *p* < 0.001, respectively) during the red-light exposure. In the blue-light condition, cortisol sensitivity was significantly negatively correlated with IL-6 sensitivity (*p* = 0.034). Overall, blue light seemed to have exerted a greater influence on cortisol and IL-6. For cortisol, this effect might be moderated by chronotype. Additionally, cortisol and IL-6 seem to interact under light exposure. However, these effects were mixed and could not be found consistently across mean secretion, AUCg and AUCi.

## 1 Introduction

The circadian rhythm refers to the regulation of the sleep-wake rhythm as well as the synchronization of a variety of other physiological and hormonal processes over a period of 24 h.

The endocrine system is among those shaped by circadian rhythmicity (e.g., Haus, 2007; Oster et al., 2017). As part of the endocrine system, cortisol in particular follows a distinct circadian rhythm in the average adult (e.g. Logan and McClung, 2019; Oster et al., 2017), characterized by an increase in the first 30–40 min after waking up and a subsequent peak, termed the cortisol-awakening response (CAR; Bowles et al., 2022; Clow et al., 2004, 2010; Stalder et al., 2016; Wilhelm et al., 2007). Approximately 40–45 min upon awakening, cortisol begins to ebb and continues to do so over the course of the day (e.g., Logan and McClung, 2019; Stalder et al., 2016).

Exposure to light constitutes an important regulating factor for circadian rhythmicity (Clow et al., 2010). Via retinal projections, light can influence the hypothalamic suprachiasmatic nucleus (SCN), the body's central circadian pacemaker, in turn shaping cortisol secretion from the adrenal cortex (Dickmeis, 2009; Ishida et al., 2005; Jung et al., 2010; Ulrich-Lai et al., 2006; Scheiermann et al., 2013). A certain subgroup of melanopsin-expressing retinal ganglion cells (mRGCs) with peak sensitivity at 480 nm, i.e., in the short-wave (blue) light spectrum (Al Enezi et al., 2011; Berson et al., 2002; Dacey et al., 2005; Hankins et al., 2008; Lucas et al., 2014; Tu, 2005; Vetter et al., 2022) provide this circadian system with non-visual information on light (Berson et al., 2002; Brainard et al., 2001; Hattar et al., 2002, 2003; Oster et al., 2017; Provencio et al., 2002). However, research on the influence of light of different wavelengths on cortisol has remained inconclusive: One the one hand, Figueiro and Rea (2012) found an increase in cortisol after an 80-min exposure to blue light (470 nm (nm)) when compared to dim light (<5 lux (illuminance)). Furthermore, in two sleep laboratory studies, Petrowski et al. observed an increased CAR (2019b) and increased cortisol (2020a) after 1 h of blue-light exposure (470–480 nm) compared to red-light exposure (635 nm). On the other hand, Petrowski et al. (2020b) did not obtain a difference in cortisol levels post-exposure to blue light (470–480 nm) versus red light (635 nm) in another sleep laboratory study.

The glucocorticoid system communicates with the immune (e.g., inflammatory) system in a bidirectional manner (Cain and Cidlowski, 2017; Silverman and Sternberg, 2012), with glucocorticoids hindering the synthesis of proinflammatory factors and bolstering anti-inflammatory mediators (Scheiermann et al., 2013). Thus, glucocorticoids undertake anti-inflammatory tasks such as the modulation of cytokine production and the transmigration of leukocytes (Coutinho and Chapman, 2011; Cruz-Topete and Cidlowski, 2015). As such, the pro-inflammatory cytokine IL-6 is typically suppressed by cortisol levels (Del Giudice and Gangestad, 2018; Petrovsky, 2001; Silverman and Sternberg, 2012).

These anti-inflammatory properties of glucocorticoids are not always effective, as glucocorticoids have been observed to have pro-inflammatory effects in response to acute stress (Cain and Cidlowski, 2017; Cruz-Topete and Cidlowski, 2015; Del Giudice and Gangestad, 2018; Sorrells and Sapolsky, 2007). Furthermore, IL-6 can raise cortisol levels by activating the HPA axis (Cain and Cidlowski, 2017; Del Giudice and Gangestad, 2018; Steensberg et al., 2003) and cannot be considered exclusively pro-inflammatory (e.g., Del Giudice and Gangestad, 2018). As such, the cytokines seem to undertake anti-inflammatory tasks (DeRijk et al., 1997) through a different signalling pathway as well (Scheller et al., 2011). Cortisol and IL-6, thus, interact in a more complex manner than a simple suppression of the latter by the former, dependent on circumstances.

This glucocorticoid-immune-circuit is also set in motion by circadian cues of light and darkness (Cain and Cidlowski, 2017). Contrary to cortisol, empirical results on diurnal rhythmicity of IL-6 have been rather sparse and diverse. Several publications indicated higher levels of IL-6 in humans during nighttime sleep (Cutolo and Straub, 2008; Dickstein and Moldofsky, 1999; Gudewill et al., 1992; Lange et al., 2010; Moldofsky, 1994; Petrovsky, 2001; Vgontzas et al., 2005), while others reported a peak in IL-6 levels after wake-up or in the morning (Bollinger et al., 2010; Born et al., 1997; Sarkar et al., 2021; Vgontzas et al., 2002). Methodological differences in these studies present a potential source for divergent findings and call for a standardized approach in a larger sample. The presented findings, however, do infer that the time frame after wake-up is of particular interest in the context of circadian fluctuations of IL-6, especially in view of the well-established CAR.

Concerning circadian rhythmicity in IL-6, evidence on the impact of light irradiation on IL-6 remains likewise inconclusive and short in number (Tähkämö et al., 2019; Petrowski et al., 2019b). One animal study investigated mice in which sepsis had been induced (Lewis et al., 2018). Subsequently, the rodents were exposed to bright, blue light (442 nm, 1400 lux), standard room lighting (fluorescent white light at 400lux) or red light (617 nm, 1400 lux; Lewis et al., 2018) for 24 h. Here, the cytokine IL-6, as one measure of systemic inflammation induced by sepsis, was significantly reduced after blue-light exposure, when compared to standard room lighting or red light. Furthermore, Xavier et al. (2010) found that in rats with collagenase-induced tendinitis exposure to low-dose LED light (880 nm) led to a reduction in inflammatory cells as well as mRNA expression of IL-6, amongst other parameters. In human cell cultures, light with a correlated color temperature of 7378 K (K) induced an upregulation of IL-6, whereas 2954 K did not (Shen et al., 2016). Additionally, ten appendicitis patients showed a greater reduction in post-operative IL-6 concentrations after 18–24 h of irradiation with bright blue light (1400 lux and peak 442 nm) compared to standard hospital lighting (nm and lux not specified; Lewis et al., 2018).

Thus, exposure to different types of light seems to influence the inflammatory system and likely its circadian rhythmicity, although the direction of the effect remains unclear. Neither is the interplay of the immune and glucocorticoid systems yet fully understood. Hence, this study investigated the effects of irradiation with light of different wavelengths (red vs. blue light) on the cytokine IL-6 as part of the inflammatory immune system. Exploratorily, the interplay of cortisol and IL-6 in the context of blue vs. red light exposure after wake-up was analyzed.

Hypothesis: Based on the study design applied and the literature provided above (Lewis et al., 2018; Petrowski et al., 2019b, 2020a), higher concentrations of cortisol and lower concentrations of IL-6 levels can be expected when participants are exposed to blue light as opposed to red light.

For the exploratory analyses, it was speculated that cortisol and IL-6 secretion were negatively linked.

## 2 Method

### 2.1 Participants

Only people who identified as male were asked to participate in this particular study. This was done in order to gauge how light affects cortisol and IL-6 concentrations uninfluenced by any moderating effects of hormonal variances. As the interplay between cortisol and IL-6 was investigated exploratorily, a potential influence of hormonal variances was to be ruled out for these analyses as well. Because age has been linked to cortisol secretion (see e.g., Law and Clow, 2020), only participants aged 18 to 35 were invited to participate in this study. Recruitment and testing of the participants occurred between October 2019 and March 2020 at the Johannes Gutenberg University in Mainz, Germany, with recruiting conducted online and via flyer advertisements. The participants received 50 Euros as compensation. Volunteers were considered eligible for participation only after successful telephone screening for potential exclusion criteria, which were defined based on factors known to impact HPA-axis activation or its reactivity. These included: Color blindness, acute and chronic illnesses, such as auto-immune diseases, coronary heart disease, disorders with chronic inflammation, metabolic disorders and blood disorders, as well as psychological disorders and allergies. Furthermore, use of psychoactive drugs and smoking of more than ten cigarettes per day excluded volunteers from participation. In addition, obesity (BMI ≥30 kg/m^2^) has been associated with altered cortisol secretion (see e.g., Herhaus and Petrowski, 2018; Herhaus et al., 2020). To control for a potential influence on cortisol with obese individuals or those bordering on obesity, a BMI of >27 kg/m^2^ was chosen as a criterium for exclusion. Prior to testing, each participant provided written informed consent. Additionally, participants were instructed not to consume any food or beverages apart from water in the 3 h before arrival at the laboratory.

### 2.2 Procedure

The study employed a sleep laboratory setting with a between-subjects experimental design, the independent variable being the light condition (red light vs. blue light)*.* This sleep laboratory setting ensured standardization across all participants and the elimination of all previous light on the system prior to the targeted exposure. To eliminate a potential bias, a computer algorithm randomly assigned participants to a light condition, with *N* = 35 participants in the red-light and *N =* 36 in the blue-light condition. To analyse blood cortisol and cytokine IL-6 as the central biological outcome measures in serum, blood samples were collected intravenously (IV) across the post-awakening period.

Testing took place at the sleep laboratory of the university after wake-up the following day. The participants were asked beforehand, via e-mail, to refrain from alcohol and any strenuous physical activity or exercise on the days leading up to testing as well as on subsequent mornings. Upon arrival, they were introduced to the testing procedure, tested for color vision and equipped and fitted with the motion sensor. They then completed a set of questionnaires which included the Perceived Stress Scale (PSS; Cohen et al., 1983), the Trier Inventory for Chronic Stress (TICS; Schulz and Schlotz, 1999), the German version of the Morningness - Eveningness Questionnaire (D-MEQ; Griefahn et al., 2001) and the Pittsburgh Sleep Quality Index (PSQI; Buysse et al., 1989). A detailed description of these questionnaires can be found in section 2.5.

**Fig. 1:**
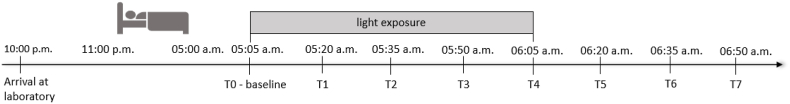
Study design, describing testing process from participants' arrival the evening prior to the last blood sample drawn. Time frames for time in bed and light exposure as well as measurement points for blood samples are depicted.

In order to rule out a potential effect on stress levels, the IV catheter was placed the evening before testing (between 10:30 and 11:00 p.m.). The participants went to bed at 11:00 p.m. in a darkened room and were awakened at 5:00 a.m. the next morning. This comparatively early wake-up time was agreed upon to minimize the probability of participants waking up prior to the standardized wake-up, inducing the CAR before it could be tested (see also Stalder et al., 2016). This approach also corresponds to an early work schedule (Figueiro and Rea, 2012). Still, any potential prior wake-up as well as sleep quality were monitored with actigraphy. Additionally, participants were instructed to wear dark sunglasses for bathroom breaks, both in the night and after wake-up. The first blood sample was taken directly upon wake-up, followed by seven additional samples at intervals of 15 min. Allotted light exposure began 5 min after wake-up and lasted 60 min, after which the participants sat in a dimly lit room (<3 (lux)) for the remaining three blood samples. The experimental procedure was completed by 7 a.m. (see Fig. 1 for study design).

### 2.3 Light conditions

For light irradiation, two half Ulbricht spheres that were indirectly illuminated by LEDs, positioned equally around the opening on the inside, were used. To ensure homogeneous illumination of the participants’ retinas, the LEDs were covered with a spectral selective diffusor. They were powered by electrical DC-dimming and controlled via computer (USB to DMX Controller). Light irradiation was created simultaneously by setting narrow-band LEDs of blue (201 lux; peak wavelength 470–480 nm; 4.26E+14 photons) or red (235 lux; peak wavelength 635 nm; 4.26E+14 photons) light, where the illuminance level was a result of the two light conditions being adjusted to the same number of photons.

Light irradiation occurred in a darkened room, with stray light levels at less than 1 lux (at the eye). Before and after each light exposure, lux was measured at eye-level using an illumination meter. The participants were seated in front of the light sources with chins resting on a chinrest, their faces thus reaching into a half-sphere (2PI-Geometry), with eye-level ensured to be consistent across all participants.

### 2.4 Biological markers: blood cortisol and cytokines

Biological markers were determined in serum. Upon the scheduled 5:00 a.m. wake-up, the first blood sample was taken, followed by seven more samples at intervals of 15 min. Blood samples were collected using Serum-Gel-Monovette® (Sarstedt, Nümbrecht, Germany) and left to coagulate for 30 min at room temperature. Subsequently, the blood samples were centrifuged for 10 min at 20 °C and 2500xG RCF. The serum was stored at −80 °C until assayed for cortisol and cytokine levels.

Serum cortisol concentrations were obtained by applying the Solid Phase Antigen Linked Technique (SPALT) to a commercially available radioimmunoassay kit with the LIAISON-Analyzer® (DiaSorin, S. p.A., Italy). The lower detection limit of this assay method is 0.43 nmol/l. Serum cytokine levels were determined with highly-sensitive ELISA enzyme-linked immunosorbent assays (IBL International GmbH, Germany), the detection limit being <0.08 pg/mL serum.

A Motionlogger® Watch and the MotionloggerWatchWare (Ambulatory Monitoring Inc., Ardsley, USA) were used to monitor the participants’ activity and rest phases as well as times awake during the night. Actigraphy data was also used to establish whether participants had woken up shortly before the allotted 5 a.m. wake-up (e.g., Stalder et al., 2016), which might have induced a premature initiation of the CAR. To eliminate a bias potentially introduced by this, awakening was defined as the transition from sleep to wake-up in accordance with the UCSD sleep algorithm (implemented in the AW2.7 software) applied to the collected PIM-data. The algorithm uses the weighted sum of all activity in a given 7-min interval except for the minute to be scored, i.e., the 4 min preceding and the two following it. When that value falls below 1, the respective minute is scored as sleep, and above 1, as being awake (see Jean-Louis et al., 2001). The data of participants with a value above 1 in the hour between 4 a.m. and 5 a.m. was subsequently excluded from further analysis. Accordingly, three participants had to be excluded from the study.

### 2.5 Instruments

As mentioned above, the interplay between cortisol and IL-6 is subject to change depending on factors such as acute stress. The well-validated (see e.g., Lee, 2012; Petrowski et al., 2019a) questionnaires pertaining to subjective stress in this study were the PSS and the TICS. The PSS is a 10-item, 5-point Likert-Scale asking respondents about their stress-related thoughts and feelings over the previous month (Cohen et al., 1983). A short of version of the TICS, the Short Screening Scale for Chronic Stress, was employed (SSCS; as described in Petrowski et al., 2019a). The SSCS is a 12-item, 5-point Likert-Scale on which respondents indicate their perceived stress levels during the previous three months. Participants' chronotype was assessed using the D-MEQ (Griefahn et al., 2001), a validated German language version of the Morningness-Eveningness Questionnaire (MEQ; Horne and Östberg, 1976). The D-MEQ is a 19-item questionnaire in which a higher score (59 and above) indicates a greater tendency towards morningness, while a lower score (41 and below) a greater tendency towards eveningness. A score in between indicates neutral chronotypes. Participants’ sleep quality across the past four weeks was assessed using the PSQI (Buysse et al., 1989). The PSQI employs different question styles (open, Likert) to assess sleep quality across different domains, e.g., average time slept during the past four weeks (open question) or amount times having difficulties staying awake during the day (Likert-scale).

### 2.6 Statistical analysis

All major statistical analyses were conducted in jamovi Version 2.2.5.0 (The jamovi project, 2021). Outlying values of more than three standard deviations above or below the mean for each measurement point and light condition were deleted. As IL-6 values were not normally distributed, they were logarithmised with the function ln+2 (+2 in order to obtain all positive values). Then, independent sample *t*-tests were applied to compare baselines between the two light conditions. In total, 19 cortisol (3,5%) and 10 IL-6 values (1,8%) were missing. As mixed linear models offer a more reliable way of conducting analysis in datasets with missing values (see e.g., Seltman, 2018), two mixed linear models for cortisol concentrations and IL-6 levels as dependent variables were subsequently calculated with the jamovi module GAMLj. In each, the two factors analyzed were light condition (red versus blue) and time (repeated measurements from T0 to T7; Fig. 1). Initially, recent chronic stress as indicated by participants on the PSS and the TICS was introduced to the mixed models as covariates. They did not prove significant however and were thus removed from the model again. As chronotype has been implicated in the level of cortisol expression during the CAR (Fries et al., 2009; Petrowski et al., 2020c), chronotype as expressed in D-MEQ scores was furthermore fed into the mixed model for cortisol and, exploratorily, IL-6. It did not emerge as a significant covariate in the latter mixed model and was again removed from it. All results are reported including significant chronotypes. All mixed models including insignificant covariates can be found in Suppl. Table 1 (supplementary materials). To gain a more thorough insight into the nature of the relationship between chronotype and cortisol expression in the study population, an independent samples *t*-test comparing D-MEQ scores between light conditions as well as Pearson's *r*-coefficients of D-MEQ scores and cortisol concentrations per light condition were subsequently calculated. To track overall output and sensitivity of both cortisol and IL-6 concentrations, Holm-corrected post-hoc comparisons were calculated from the mixed model where applicable and group comparisons for area-under-the-curve statistics with respect to ground (AUC_G_) and increase (AUC_I_; Pruessner et al., 2003; Fekedulegn et al., 2007) were calculated in Microsoft Excel. Lastly, Cortisol and IL-6, AUC_G_ for Cortisol and IL-6 as well as AUC_I_ for Cortisol and IL-6 were correlated per light condition using Pearson's *r*-coefficients. This was carried out in order to explore a potential interplay of Cortisol and IL-6. The effect size measures *r*, *d* and *f*^2^ (the latter as per Cohen, 1992) are reported where applicable.

## 3 Results

The mean age of the N = 71 participants analyzed was *M* = 24.5, *SD* = 3.78 years. There was no significant difference in age between the red-light group and the blue-light group, *t* (69.0) = −0.368, *p* = 0.720, *d* = −0.09, 95% CI [–0.550, 0.381]. Mean D-MEQ scores were *M* = 49.3, *SD* = 10.8, for the red-light group and *M* = 52.0, *SD* = 8.03 for the blue-light group. A significant group difference between light conditions in mean D-MEQ scores, *t* (566) = 3.44, *p <* 0.001, *d* = 0.29, 95% CI [0.123; 0.455], was found.

### 3.1 Cortisol

**Fig. 2:**
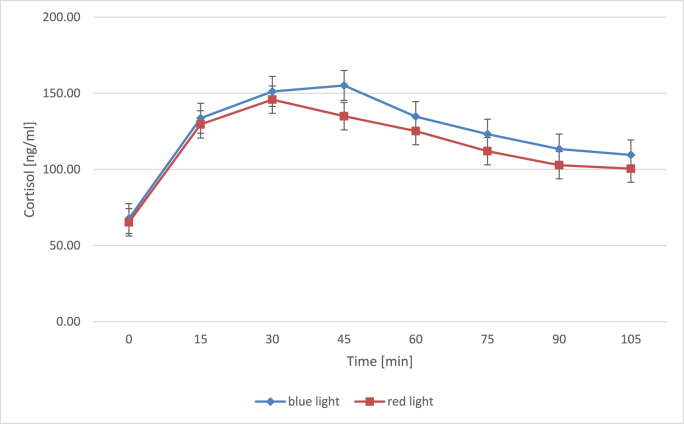
Mean (±*SE*) cortisol levels in nanograms per milliliter across measurement points for blue- and red-light conditions. Vertical beams indicate standard errors. Timepoints in minutes correspond to measurement points T0 to T7 in Fig. 1. (For interpretation of the references to color in this figure legend, the reader is referred to the Web version of this article.)

Means, standard deviations as well as post-hoc comparisons of cortisol concentrations for both light conditions are depicted in Table 1 and cortisol levels across measurement points in Fig. 2. No significant difference regarding the baseline measurement could be observed between the groups, *t* (66) = 0.49, *p =* 0.627, *d* = 0.12, 95% CI [–0.365; 0.595]. A mixed model was calculated analyzing the effects of time, light condition and chronotype as a covariate on mean cortisol concentrations. This mixed model revealed a highly significant main effect for time, *F* (7 463.7) = 75.54, *p* < 0.001, *f*^2^ = 0.38. Comparisons between each measurement point and the one succeeding it (Table 1) reveals that cortisol increased significantly between the first sample directly upon wake-up and the second samples 15 min later for both light conditions. AUCi values indicated an overall increase of cortisol values over time for both conditions (Table 2).

**Table 1:** Means and standard deviations for cortisol levels across measurement points by light condition.

Time	Red light			Comparison^a^		Blue light			Comparison^a^	
	*n*	*M*	*SD*	*p*_*uncorr*_	*p*_*holm*_	*n*	*M*	*SD*	*p*_*uncorr*_	*P*_*holm*_
0	32	65.3	37.4			35	69.5	35		
+15	33	130	45.2	<0.001	<0.001	34	134	39.5	<0.001	<0.001
+30	34	146	50.1	0.019	0.985	34	151	34.5	0.003	0.193
+45	34	135	52.9	0.070	1.000	34	151	41.6	0.928	1.000
+60	34	125	57.1	0.104	1.000	35	140	48.4	0.016	0.841
+75	34	112	47	0.028	1.000	34	123	42.3	0.026	1.000
+90	34	103	44.7	0.127	1.000	34	113	46.9	0.030	1.000
+105	33	101	42.9	0.541	1.000	33	106	38.1	0.478	1.000

Note.

All corrected comparisons apply Holm correction.

a Comparisons are to the preceding time point each (+15 to 0, +30 to +15 etc.).

**Table 2:** Means and standard deviations for AUCg and AUCi values in the red- and blue-light group.

	Red light				Blue light			
	AUCg		AUCi		AUCg		AUCi	
	*M*	*SD*	*M*	*SD*	*M*	*SD*	*M*	*SD*
Cortisol	12277	4151	5046	3225	13202	3469	6219	2985
IL-6	330	73.9	0.263	64.9	352	66.6	1.85	67.7

*Note*. IL-6 means were logarithmised applying a ln+2 formula.

There was a significant main effect for chronotype as a covariate, *F* (1 64.4) = 4.62, *p* = 0.035, *f*^2^ = 0.38, but no significant interaction between chronotype as a covariate, light condition and mean cortisol concentrations, *F* (1 449.5) = 0.79, *p* = 0.594, *f*^2^ = 0.38. Furthermore, no significant main effect for light condition, *F* (1 64.5) = 0.60, *p* = 0.443, *f*^2^ = 0.38, or significant interaction between light condition and mean cortisol concentrations was present either, *F* (7 463.7) = 1.01, *p* = 0.427, *f*^2^ = 0.38. Significant correlations between cortisol concentrations and chronotype emerged, both for the blue-light group, *r* = 0.13, *p* = 0.031, 95% CI [0.012; 0.242], and for the red-light group, *r* = 0.26, *p* < 0.001, 95% CI [0.147; 0.370]. Means and standard deviations of AUC_G_ and AUC_I_ values for cortisol are listed in Table 2. Both AUC_G_, *t* (547) = 2.84, *p* = 0.050, *d* = 0.24, 95% CI [0.074, 0.411], and AUC_I_, *t* (547) = 4.42, *p* < 0.001, *d* = 0.38, 95% CI [0.207, 0.548], were significantly higher in the blue-light group.

### 3.2 IL-6

**Fig. 3:**
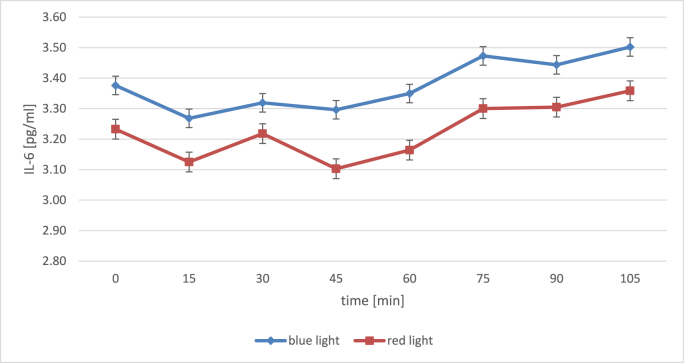
Mean (±*SE*) IL-6 levels in picograms per milliliter across measurement points for blue- and red-light conditions, logarithmised with ln+2. Vertical beams indicate standard errors. Timepoints in minutes correspond to measurement points T0 to T7 in Fig. 1. (For interpretation of the references to color in this figure legend, the reader is referred to the Web version of this article.)

Table 3 provides means, standard deviations and post-hoc comparisons for IL-6 levels in both groups, while Fig. 3 illustrates IL-6 concentrations across measurement points. There was no significant difference regarding the baseline measurement, *t* (66) = 0.82, *p =* 0.414, *d* = 0.20, 95% CI [–0.280; 0.677]. A mixed model was calculated analyzing the effects of time and light condition on mean IL-6 concentrations. First and foremost, time, *F* (7 473.1) = 3.32, *p* = 0.002, *f*^2^ = 0.03, showed a significant main effect. Here, Holm-corrected comparisons between each measurement point and the one succeeding it (Table 3) did not reveal significant changes in mean IL-6 concentrations from one sample to the next in either light condition. However, AUCi values described an overall increase of IL-6 values over time for both conditions (Table 2).

**Table 3:** Means and standard deviations for IL-6 levels across measurement points by light condition.

Time	Red light			Comparison^a^		Blue light			Comparison^a^	
	*n*	*M*	*SD*	*p*_*uncorr*_	*p*_*holm*_	*n*	*M*	*SD*	*p*_*uncorr*_	*p*_*holm*_
0	32	3.23	0.96			35	3.41	0.79		
+15	34	3.12	0.79	0.447	1.000	35	3.29	0.90	0.281	1.000
+30	34	3.22	0.84	0.568	1.000	35	3.32	0.81	0.781	1.000
+45	35	3.10	0.77	0.387	1.000	35	3.35	0.78	0.818	1.000
+60	35	3.16	0.75	0.580	1.000	35	3.40	0.77	0.614	1.000
+75	35	3.30	0.77	0.218	1.000	34	3.52	0.74	0.170	1.000
+90	35	3.30	0.78	0.966	1.000	34	3.44	0.86	0.457	1.000
+105	34	3.36	0.79	0.455	1.000	33	3.54	0.74	0.237	1.000

*Note:* Means were logarithmised applying a ln+2 formula prior to and for mixed model analysis.

All corrected comparisons apply Holm correction.

a Comparisons are to the preceding time point each (+15 to 0, +30 to +15 etc.).

There was no significant main effect for light condition, *F* (1 68.9) = 1.47, *p* = 0.230, *f*^2^ = 0.03. The interaction term for light condition and mean IL-6 concentrations was not significant, *F* (7 466.1) = 0.18, *p* = 0.989, *f*^2^ = 0.03. Means and standard deviations of AUC_G_ and AUC_I_ values for IL-6 can also be found in Table 2. AUC_G_ in the blue-light group was significantly higher than AUC_G_ in the red-light group, *t* (556) = 3.56, *p* < 0.001, *d* = 0.30, 95% CI [0.133; 0.469]. There was no significant difference in AUC_I_ values between the light conditions, *t* (556) = 0.28, *p* = 0.777, *d* = 0.02, 95% CI [–0.142; 0.190]. Cortisol and IL-6 concentrations were not significantly correlated in the blue-light condition, *r* = −0.01, *p* = 0.914, 95% CI [–0.124; 0.111], but in the red-light group, *r* = −0.14, *p* = 0.021, 95% CI [–0.021; 0.257]. Correlations between Cortisol and IL-6 for AUC_G_ and AUC_I_ values per light condition are depicted in Table 4.

**Table 4:** Pearson's *r-*coefficients between Cortisol and IL-6 AUCg and AUCi values per light condition.

	AUCg				AUCi			
	Red light		Blue light		Red light		Blue light	
	*r*	*p*	*r*	*p*	*r*	*p*	*r*	*p*
Red light	−0.23 [−34, −0.11]	**<**0.001**	–	–	0.01 [−0.11, 0.13]	0.860	–	–
Blue light	–	–	−0.04 [−0.16, −0.07]	0.461	–	–	−0.13 [−0.24, −0.01]	0.034*

*Note*. IL-6 means were logarithmised applying a ln+2 formula. Significant correlations indicated in bold.

95% CI indicated in square brackets.

*: *p <* 0.05. **: *p* < .001. 95% CI indicated in square brackets.

## 4 Discussion

Over a period of 24 h, the alternation of sleep and wakefulness as well as a variety of other biological processes are synchronized. This synchronization is referred to as the circadian rhythm, with light cues constituting a major influencing factor (Clow et al., 2010). While the immune system is recognized as regulated by circadian rhythmicity (e.g., Oster et al., 2017; Scheiermann et al., 2013), there are open questions regarding the impact of different kinds of light exposure on specific immune parameters such as the inflammatory marker IL-6. Thus, this study aimed to shed further light on this question while simultaneously exploring a potential interplay with the stress hormone cortisol.

In the present study, post-hoc comparisons revealed a significant rise in cortisol levels in both light groups from the baseline measurement (5 min after wake-up) to 15 min later, indicative of the occurrence of a CAR. Contrary to previously reported increased cortisol levels in response to blue light exposure (Petrowski et al., 2019b, 2020a), no differences between mean cortisol concentrations depending on light condition could be obtained in the current study. The present findings are in line with Figueiro and Rea (2012) and Petrowski et al. (2020b) who did not obtain a difference in average cortisol concentrations depending on exposure to blue or red light. Nevertheless, AUC calculations indicated a greater overall cortisol output (AUC_G_) as well as greater sensitivity (AUC_I_; see e.g., Fekedulegn et al., 2007) of cortisol in the blue-light condition. Chronotype was significantly correlated with cortisol concentration and proved a significant covariate in the mixed model. This replicates previous findings of earlier chronotypes exhibiting greater cortisol secretion during the CAR (Fries et al., 2009; Petrowski et al., 2020c). In this study, morning chronotypes happened to be sorted significantly more often into the blue light condition. Hence, this chronotype distribution might have raise AUC_G_ scores in the blue-light condition and led to skewed results.

Regarding pro-inflammatory cytokines, the present study showed a significant main effect of time for average IL-6 concentrations, with mean AUC_I_ values indicating an overall increase in both light conditions. However, as neither the interaction in the mixed model nor the comparison between mean AUC_I_ values was significant, IL-6 was not more sensitive to (i.e., shaped in its course by) either red or blue light in particular. Whereas no difference in average IL-6 concentrations between exposure to red or blue light was shown, a greater overall IL-6 output (AUC_G_) could be observed in the blue-light condition. Hence, these findings do not replicate those brought forth by Lewis et al. (2018).

Concerning the interplay of cortisol and IL-6, a greater average cortisol concentration correlated with a lower average IL-6 concentration for participants exposed to red light. Additionally, a greater overall cortisol secretion (AUC_G_) was linked to lower overall IL-6 secretion in this group. The cortisol sensitivity (AUC_I_, change of over time) was, however, not linked to the sensitivity of IL-6 in the red-light condition. In the blue-light condition, exactly opposing results were obtained: Here, the mean and overall cortisol output was not related to the mean and overall IL-6 concentrations. However, cortisol and IL-6 sensitivity were linked significantly. More specifically, a greater overall increase in cortisol was associated with a greater overall decrease in IL-6, and vice versa. As cortisol typically produces anti-inflammatory effects (Del Giudice and Gangestad, 2018; Petrovsky, 2001; Silverman and Sternberg, 2012), decreasing concentrations of the primarily pro-inflammatory cytokine IL-6 (e.g., Del Giudice and Gangestad, 2018) are in line with the mode of action of increasing cortisol concentrations. In this study, this interplay was exclusively observed in the blue light condition, even though overall secretion was not related between the two parameters. While the sensitivity of cortisol and IL-6 was not significantly correlated in the red-light condition, their greater total cortisol output was accompanied by lower total IL-6 output. Different types of light might serve different purposes in the interplay between cortisol and IL-6 depending on environmental challenges. In preparation for a normal upcoming day or night, so at times when natural light appears warmer and more orangey (e.g., Pastilha and Hurlbert, 2022), cortisol might be required to influence IL-6 output (or vice versa) more greatly. Similarly, during the day, when natural lighting appears bluer (e.g., Pastilha and Hurlbert, 2022), cortisol and IL-6 sensitivity might need to be able to interact more readily to meet daily challenges when actually out and about in the world. Of course, this is mere speculation at this point.

There are both strengths and limitations to this study. The test protocol was highly standardized, with the participants sleeping at the lab and awakened at a standardized time. Their sleep was monitored using actigraphy and any participants who woke up prior to the scheduled wake-up time were excluded from analysis. Moreover, prior to the experimental light irradiation, any non-experimental light sources were controlled by keeping the room where participants were staying completely dark, with values below one lux. This ensured any effects of light exposure to be clearly traced back to the experimental light exposure. Additionally, precise mapping of CAR trajectories was enabled by selecting standardized measurement points considered best for this purpose (see e.g., Stalder et al., 2016), its adherence ensured by the personnel conducting the testing. As to limitations in this study, in order to observe the potential influence of light of different wavelengths on the CAR and IL-6 concentrations independently of any moderating effects of hormonal variances, only people who identified as male participated in this study. However, sex differences in immune responses are a known phenomenon (e.g., Fish, 2008). While evidence on sex differences in IL-6 expression in particular is inconsistent (Chapman et al., 2009; Lockwood et al., 2016), it is nevertheless of great importance to include female participants in future research efforts on the effects of light exposure on IL-6 and its interplay with cortisol. Additionally, it should be pointed out that effect sizes for the mixed model of light conditions and IL-6 were small. It might thus be that the sample analyzed was simply not powered enough to detect any existent differences in the mixed model.

Future research aimed at the influence of various types of light on the CAR might implement a within-subject design or mixed between-/within-subject. Intraindividual differences in CAR expression across different days have been observed (e.g., Stalder et al., 2016), and both studies by Petrowski et al., in which blue light led to significantly higher cortisol expression (2019b, 2020a), were conducted in a within-subject design. Thus, a study design encompassing two or more CAR trajectories per person might better encompass these differences and the role they may play in the effect of various types of light on the CAR.

Regarding the interplay of cortisol and IL-6, the interaction of these two parameters during different times of the day and factors that might shape this interplay should be investigated further. While chronotype was not a significant covariate in the relationship between light exposure and IL-6 levels in this study, chronotype did play a significant role for cortisol. Subsequent groups of different chronotypes should be investigated for the interplay of cortisol and IL-6. Furthermore, as acute stress has been observed to provoke pro-inflammatory actions in cortisol (Cain and Cidlowski, 2017; Cruz-Topete and Cidlowski, 2015; Del Giudice and Gangestad, 2018; Sorrells and Sapolsky, 2007), studying pro-inflammatory actions and the interplay after an acute stressor in the morning might lead to further insights.

In conclusion, the present results replicate findings on chronotype-specific cortisol expression during the CAR. While there was no difference in average cortisol concentrations between conditions, the overall cortisol output and sensitivity was greater in the blue-light condition. Regarding the pro-inflammatory cytokine response, IL-6 generally increased over time. Mean IL-6 concentrations and IL-6 sensitivity did not differ between exposure to red or blue light after wake-up. However, total IL-6 secretion was greater in the blue-light condition. All in all, cortisol and IL-6 seemed to have been influenced more greatly by blue light. For cortisol however, this effect might be explained, at least to some extent, by chronotype. Exploratory analyses furthermore revealed that cortisol and IL-6 appear to interact in some measure in the context of light exposure. Thus, the proposed hypothesis can only partially be confirmed, and only for cortisol. Concerning the interplay between cortisol and IL-6, a greater mean cortisol concentration and total cortisol output was linked to lower mean IL-6 concentrations and total IL-6 output for the red-light condition, with increase in cortisol related to decrease in IL-6 only for the blue-light condition. Further, more in-depth knowledge on the complex interplay of cortisol and IL-6 in different lighting conditions and the purposes this might serve for human adaption to environmental challenges is needed.

## Ethical approval and consent

The study protocol was approved by the Ethics Committee of the Medical Faculty at the Technical University of Dresden, Germany (No. #EK353092014), and the study was conducted in accordance with the Declaration of Helsinki (2013). Prior to testing, each participant provided written informed consent.

## CRediT authorship contribution statement

**Liza Mekschrat:** Writing – review & editing, Writing – original draft, Formal analysis. **Bjarne Schmalbach:** Methodology. **Nicolas Rohleder:** Resources, Formal analysis. **Katja Petrowski:** Writing – review & editing, Supervision.

## Declaration of competing interest

This research received funding from the 10.13039/501100002347Federal Ministry of Education and Research (BMBF), Germany. This study was conducted in the subproject: “Einfluss von Licht auf die hormonelle Stressverarbeitung” [influence of light on hormonal stress management] as part of the joint project: “Nicht-visuelle Lichtwirkungen” [non-visual effects of light] (Funding code: 13N13397).

## Data Availability

Data will be made available on request.
