# Supplementary material for: IL-6 after wake-up in human males: Exposure to red versus blue light and the interplay with cortisol
Source: Brain Behav Immun Health. 2024 Aug 2;40:100833. doi: 10.1016/j.bbih.2024.100833 (PMC11381833; doi:10.1016/j.bbih.2024.100833)
Supplement: Multimedia component 1 [file mmc1.docx]

| Suppl Table 1  Mixed model results for initial mixed models including insignificant covariates | | | | |
| --- | --- | --- | --- | --- |
|  |  | **Red vs. blue light** | | |
|  |  | *F*(df) | *p* | *f*^2^ |
| Cortisol | Time | 75.62 | **< .001**** | .40 |
|  | Light | 1.17 | .283 | .40 |
|  | Time*light | 1.08 | .376 | .40 |
|  | D-MEQ | 5.07 | **.028*** | .40 |
|  | PSS | 1.00 | .320 | .40 |
|  | TICS | 0.79 | .377 | .40 |
| IL-6 |  | **Red vs. blue light** | | |
|  |  | *F*(df) | *p* | *f*^2^ |
|  | Time | 3.32 | **.002*** | .05 |
|  | Light | 3.32 | .242 | .05 |
|  | Time*light | 0.16 | .993 | .05 |
|  | D-MEQ | 0.85 | .359 | .05 |
|  | PSS | 0.43 | .516 | .05 |
|  | TICS | 0.14 | .906 | .05 |
| Note. D-MEQ = German version of the morningness-eveningness-questionnaire. PSS = Perceived Stress Scale. TICS = Trier Inventory for Chronic Stress. Time denotes main effects of repeated blood measurements. Light denotes main effects of red vs. blue light. Time*light denotes interaction terms. *f*^2^ calculated from *R*^2^ for entire model. Significant *p*-values indicated in bold. * *p* < .05. ** *p* < .001. | | | | |
